# Early Institutional Experience With the Dynamic Femur Fracture (DF2) Brace for Pediatric Femoral Fractures: An Institutional Case Series

**DOI:** 10.7759/cureus.113078

**Published:** 2026-07-21

**Authors:** Rami Rajjoub, Asahi Murata, Aaron DeVilbiss, Kirsten Tulchin-Francis, Sean A Tabaie

**Affiliations:** 1 Orthopedic Surgery, Nationwide Children's Hospital, Columbus, USA; 2 Orthopedics, Nationwide Children's Hospital, Columbus, USA

**Keywords:** fracture, hip brace, pediatric femoral neck fracture, pediatric surgery, spica

## Abstract

Traditional spica casting is the standard treatment for pediatric femoral fractures in children under five years of age, but it carries well-documented limitations, including the need for general anesthesia, increased caregiver burden, and high rates of skin complications. Prefabricated functional bracing, such as the Dynamic Femur Fracture (DF2) brace (OrthoPediatrics, Warsaw, IN), has emerged as an alternative that eliminates anesthesia exposure and improves patient mobility. However, clinical data regarding the practical limitations, complication profiles, and patient selection boundaries during early adoption remain limited. This case series describes our institution's early experience with the DF2 brace to provide practical insights into patient selection and brace management. Following Institutional Review Board (IRB) approval, a case series was conducted at a single tertiary care pediatric institution. Records were reviewed for patients aged under 18 years diagnosed with a fracture requiring functional bracing treatment between January 1, 2020, and October 17, 2025. Patients were excluded if they had inadequate clinical and radiographic follow-up of less than six weeks. Demographic variables, injury characteristics, treatment parameters, radiographic time to union, and soft-tissue complications were extracted from electronic medical records into a secure database. Five pediatric patients were evaluated (four traumatic fractures and one elective postoperative reconstruction). Successful fracture healing and a return to age-appropriate activity were achieved in all four traumatic fracture cases. Open, superficial pressure injuries of the proximal lateral calf occurred in two patients, with both successfully managed in the outpatient clinic setting with local wound care. In an elective hip reconstruction case involving a 12-year-old nonambulatory patient with advanced cerebral palsy, the brace dug deeply into the groin and impinged on a pre-existing vesicostomy site, necessitating discontinuation on postoperative day 1 in favor of a straight knee immobilizer. Ultimately, the DF2 brace serves as an alternative to spica casting for acute, isolated femoral fractures in young children, providing reliable bone healing with simplified hygiene access. However, it did not eliminate the risk of pressure injuries in this series, highlighting the need for vigilant stockinette hygiene and proactive padding.

## Introduction

Femoral fractures are among the most common pediatric orthopedic injuries requiring hospitalization [1], and their management is heavily influenced by patient age, fracture pattern, and family considerations [2]. For children six months to five years old with low-energy diaphyseal fractures and less than 2 cm of shortening, the American Academy of Orthopaedic Surgeons (AAOS) recommends early spica casting or traction with delayed spica casting as standard treatment [2,3]. While spica casting reliably stabilizes fractures and yields predictable healing, it is associated with decreased patient and caregiver quality of life [4,5]. Casts are typically applied under general anesthesia, and their rigid, nonadjustable nature limits hygiene, toileting, transportation, and routine skin assessment [3,4,6,7]. Reported complications include skin breakdown in up to 28% of patients, with younger age, abuse-related mechanism, and cast duration over 40 days as significant predictors, along with soiling, pressure injuries, and the need for wedging or reapplication that add to caregiver burden and clinical follow-up [5,7-9].

Functional femoral bracing has emerged as an alternative for select pediatric femoral fractures [4,10]. The prefabricated Dynamic Femur Fracture (DF2) brace (OrthoPediatrics, Warsaw, IN) uses hinged thigh and calf shells connected by an adjustable strap system, allowing caregivers to tighten the device as swelling decreases and to remove sections for bathing and skin checks without disrupting alignment. It can typically be applied without sedation and permits more frequent skin inspection than a spica cast, potentially reducing the need for general anesthesia [4]. Prior studies have shown promising radiographic and functional outcomes with functional bracing, but most have focused on fracture healing and alignment, leaving a gap regarding early complications and variability in care protocols during initial clinical adoption [4,10].

As DF2 bracing use increases, understanding its practical limitations is essential for optimizing patient safety and outcomes. Pressure-related skin injuries, challenges with stockinette management, the loss of reduction, and variability in brace adjustment may represent underrepresented pitfalls. The purpose of this case series was to characterize clinical outcomes, complications, and practical implementation challenges associated with the early adoption of the DF2 brace for pediatric femoral fractures, providing practical insights into patient selection, brace management, and early complications for clinicians considering functional bracing as an alternative to traditional spica casting.

## Case presentation

Following Institutional Review Board (IRB) approval, a retrospective case series study was conducted to evaluate the clinical application and outcomes of the DF2 brace in pediatric patients at a single tertiary care pediatric institution between January 1, 2020, and October 17, 2025. Inclusion criteria included male or female patients under 18 years of age diagnosed with a fracture requiring functional bracing treatment. Patients were also excluded from the analysis if they did not ultimately receive a brace or if they had inadequate medical records, which were defined as a clinical and radiographic follow-up period of less than six weeks. Patient safety and risk minimization were maintained by evaluating only standard-of-care clinical follow-up records.

Case 1

**Figure 1:**
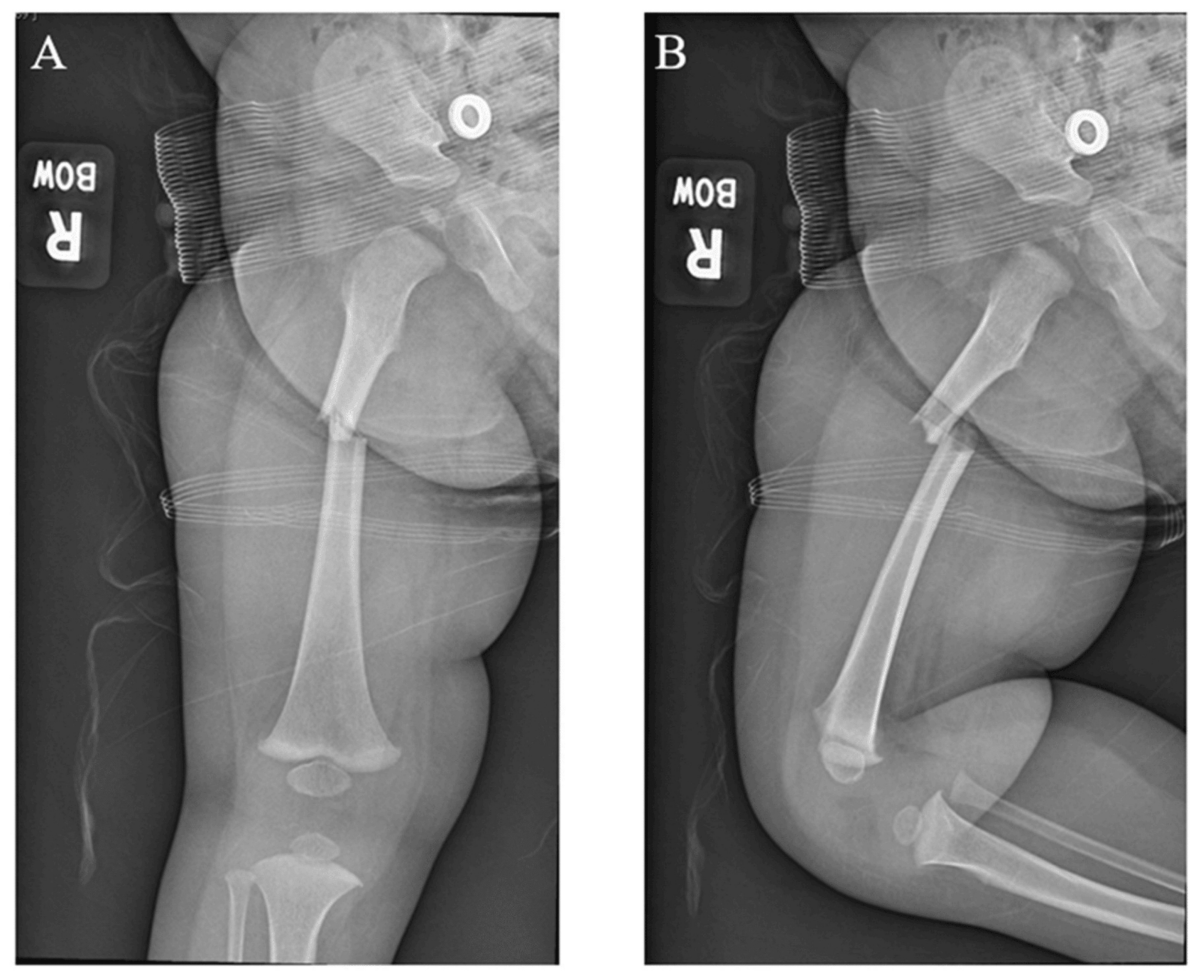
Initial imaging for case 1, including (A) AP and (B) lateral radiographs of the right lower extremity demonstrating a transverse fracture of the proximal third of the femur

Case 1 was a previously healthy 10-month-old who presented to the emergency department (ED) after sustaining a right thigh injury after an unmounted television fell onto his leg. The patient was accompanied by his parents, who were non-English-speaking. On examination, the patient demonstrated significant swelling and severe tenderness to palpation across the fracture site. The overlying skin was intact, and the neurovascular examination was normal. All compartments of the lower extremity were soft. Radiographs were obtained in the ED and revealed a closed displaced diaphyseal transverse fracture of the right femur (Figure 1A, 1B). Given the patient's age and the mechanism of injury, the patient was initially evaluated by the Child Assessment Team for non-accidental trauma, which was ruled out. His skeletal survey was negative. He was placed in a temporary long-leg posterior splint and subsequently taken to the operating room (OR) the next morning for the closed reduction and placement of the DF2 femur brace. The fracture was first held out to length for reduction, and then, the brace was applied with the hip positioned at 30° of abduction and 30° of flexion. Postreduction fluoroscopic images confirmed adequate alignment. The thigh was protected with a stockinette to avoid direct contact between the brace and skin. Distal capillary refill remained intact following brace placement. The patient was discharged home later that day after meeting all standard discharge protocols and being fitted for a car seat while in the brace.

**Figure 2:**
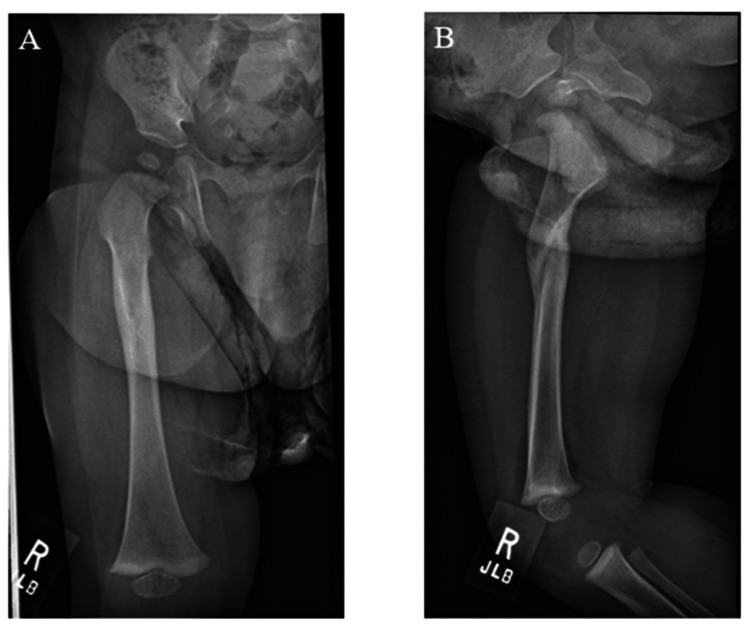
Final imaging for case 1, including (A) AP and (B) lateral radiographs of the right lower extremity demonstrating the healing of the proximal femoral diaphyseal fracture

The postoperative course was unremarkable, and the patient's pain was successfully managed with a scheduled oral analgesic regimen. Clinical and radiographic follow-up occurred at one week, two weeks, four weeks, six weeks, and three months. Radiographs obtained during the one-week postoperative period demonstrated mild shortening and displacement, which were stable during the rest of the follow-up period. The brace was adjusted and reinforced with moleskin padding along the right proximal thigh. Week-2 radiographs demonstrated early callus formation. The brace was temporarily removed to replace the stockinette and perform a skin examination, which revealed no pressure injuries or irritation. Skin examination continued to show no complications at all other follow-up intervals. The brace was removed during the six-week follow-up after radiographs demonstrated adequate healing and examination revealed tenderness to palpation (Figure 2A, 2B). At the final three-month follow-up visit, the parents were counseled and reassured about a mild delay in ambulation.

Case 2

**Figure 3:**
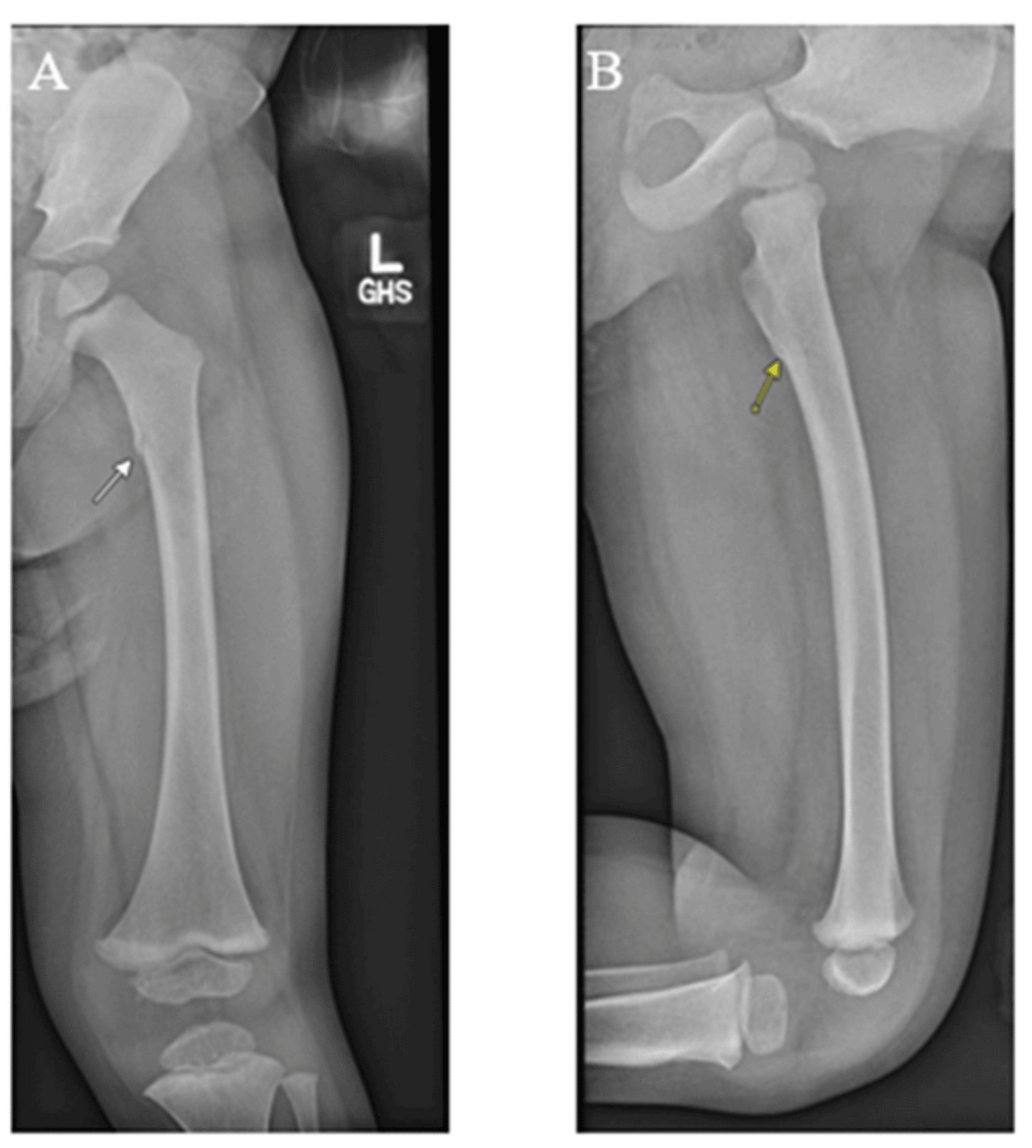
Initial imaging for case 2, including (A) AP and (B) lateral radiographs of the left lower extremity demonstrating a nondisplaced fracture of the medial cortex of the subtrochanteric proximal femur

Case 2 was a previously healthy two-year-and-four-month-old boy who presented to the ED after being unable to bear weight in the lower left extremity after falling from a set of monkey bars. On examination, the patient was mildly tender to palpation on the anterior left hip. Neurovascular examination was intact throughout the entire left extremity. Initial pelvic and hip radiographs demonstrated an intertrochanteric fracture of the left femur (Figure 3A, 3B). The patient was taken to the OR the following morning for DF2 placement under general anesthesia. The left lower extremity was held in flexion and abduction as the stockinette was placed, followed by the DF2 brace. Both the thigh and lower leg components were confirmed to be well-fitting. The brace was initially set to allow a 30° arc of motion at the hip between 60° and 90° of flexion. The family was instructed to keep the patient non-weight-bearing on the lower left extremity and to follow up in the outpatient clinic in four weeks.

**Figure 4:**
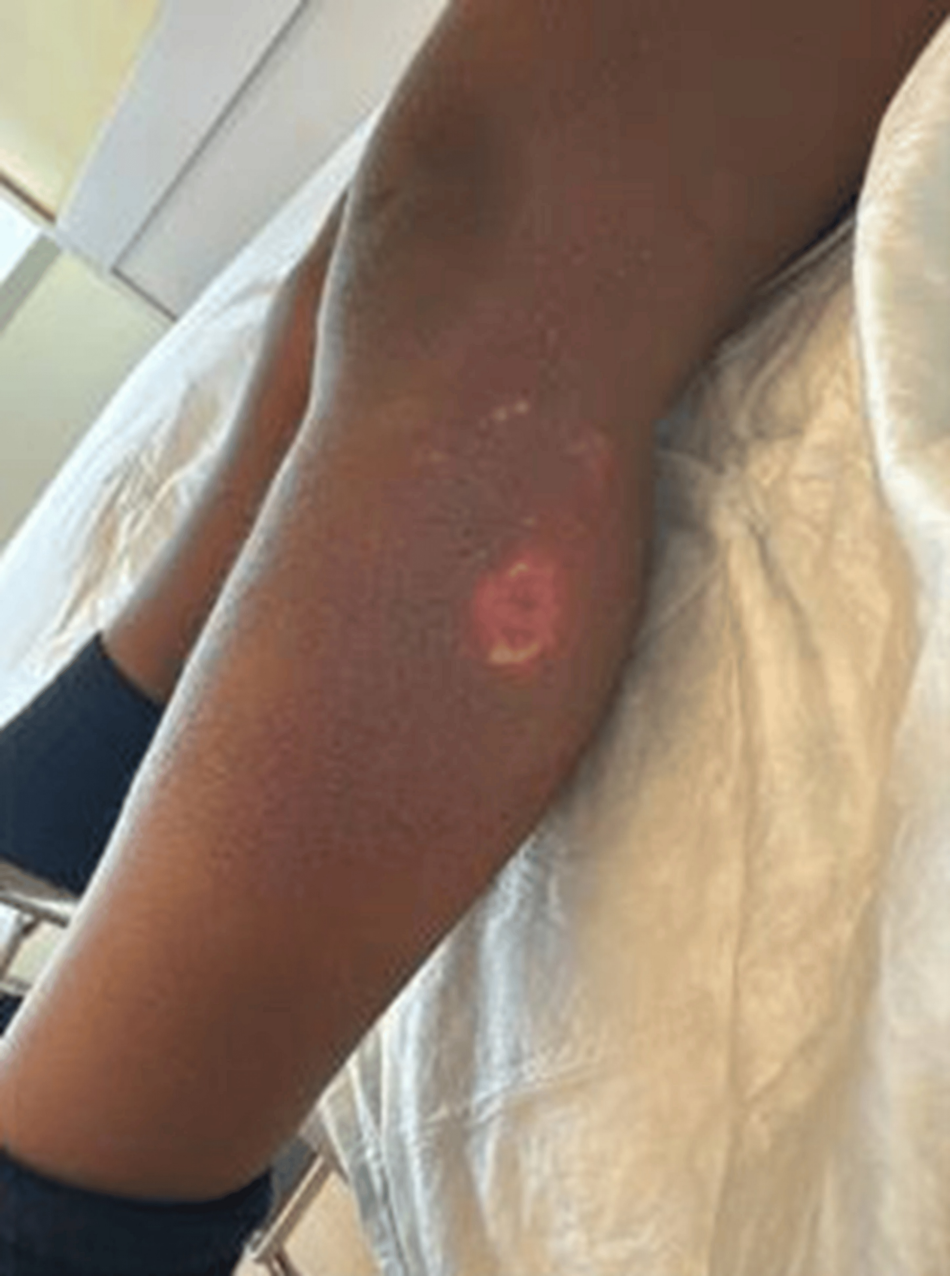
Image of case 2 at three-week follow-up that demonstrates an open, circular pressure injury measuring 2-3 cm in diameter along the proximal lateral aspects of the left calf, approximately 5 cm distal to the knee joint

**Figure 5:**
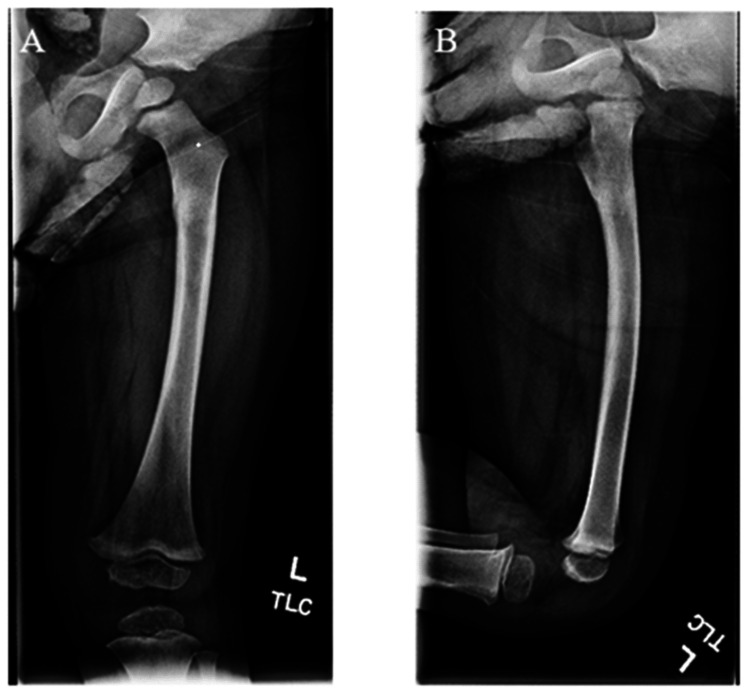
Final imaging for case 2, including (A) AP and (B) lateral radiographs of the left lower extremity demonstrating the healing of the subtrochanteric proximal femur fracture

However, the patient returned to the clinic at the three-week mark due to concerns about a superficial skin lesion. Physical examination revealed an open, circular pressure injury measuring 2-3 cm in diameter along the proximal lateral aspect of the left calf, approximately 5 cm distal to the knee joint (Figure 4). The wound exhibited a foul odor and a small amount of serosanguineous drainage. Since the patient was non-tender along the fracture site, the brace was removed at the three-week visit, and the patient was transitioned to age-appropriate activity restrictions without immobilization. The patient was also prescribed a five-day course of cephalexin for wound management, as well as antibacterial soap, Betadine, and a Mepilex silver dressing covered with Kerlix gauze until the next follow-up at the four-week mark. The patient returned for the four-week follow-up clinic visit, at which time the skin injury had demonstrated appropriate healing. Additionally, the patient was reported to be running at home without any pain. Follow-up radiographs demonstrated excellent bone healing, and the patient was discharged from continued orthopedic care (Figure 5A, 5B).

Case 3

**Figure 6:**
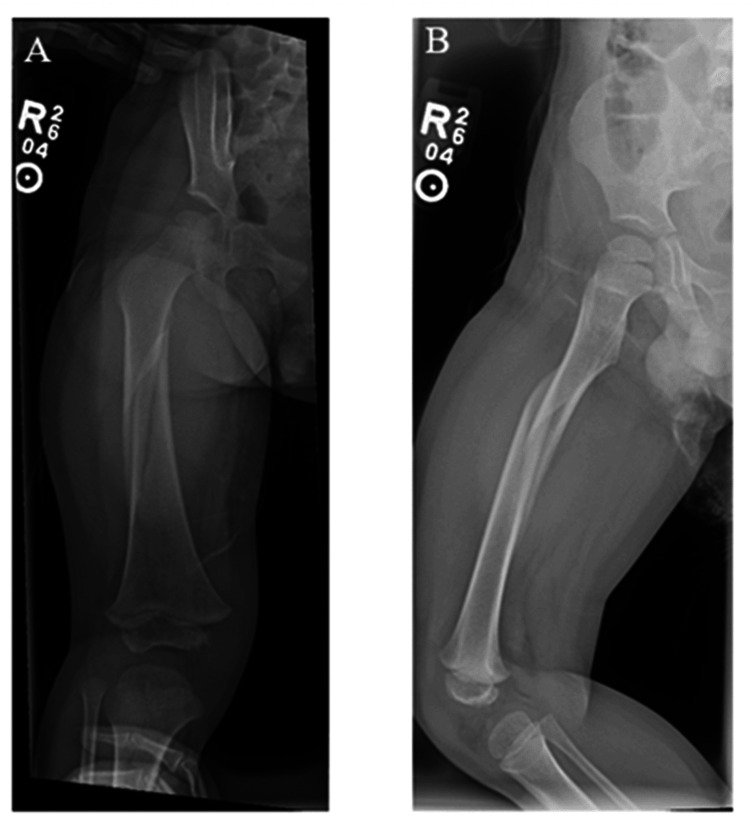
Initial imaging for case 3, including (A) AP and (B) lateral radiographs of the right lower extremity demonstrating an oblique subtrochanteric femoral shaft fracture

Case 3 was a previously ambulatory two-year-old-and-five-month-old boy with a past medical history significant for asthma and cystic fibrosis transmembrane conductance regulator (CFTR)-related metabolic syndrome who presented to the ED after falling from a bunk bed and landing on his right lower extremity. On physical examination, there was obvious deformity of the right thigh accompanied by significant soft-tissue swelling and exquisite tenderness to palpation. The neurovascular examination of the right lower extremity was intact. Radiographs of the right lower extremity demonstrated a displaced, oblique femoral shaft fracture (Figure 6A, 6B). The patient was initially stabilized with a long-leg posterior splint and taken to the OR the following morning for closed reduction and application of the DF2 under general anesthesia. The brace was applied over a protective stockinette and subsequently locked into position. The patient tolerated the procedure well and was discharged home with instructions to follow up for the two-week, four-week, and two-month periods.

**Figure 7:**
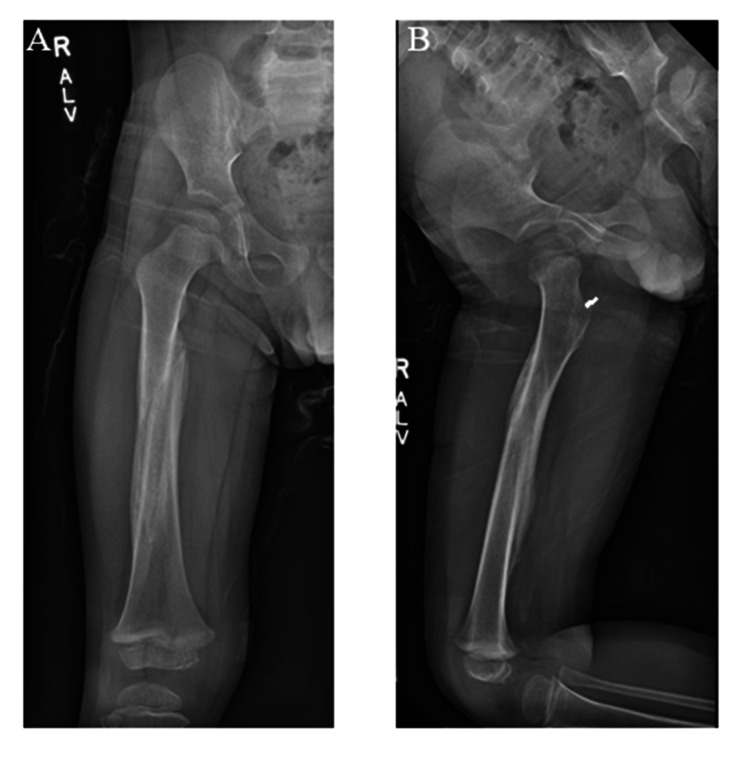
Final imaging for case 3, including (A) AP and (B) lateral radiographs of the right lower extremity demonstrating the healing of oblique subtrochanteric femoral shaft fracture

At the initial two-week follow-up visit, the patient was tolerating the brace well with no acute concerns. The brace and stockinette were removed for examination, which revealed healthy skin with no pressure ulcers or irritation. A new stockinette was applied, and the brace was reapplied for an additional two weeks. At the four-week follow-up visit, the patient had progressed to crawling in the brace with no concerns for pain. Radiographs taken at this time demonstrated early callus formation and stable alignment. Given these findings, the brace was discontinued, and the patient was allowed to transition to progressive weight-bearing as tolerated. At the two-month follow-up, the patient had resumed independent ambulation. Serial radiographs confirmed adequate bone healing, and the patient was discharged from pediatric care (Figure 7A, 7B).

Case 4

**Figure 8:**
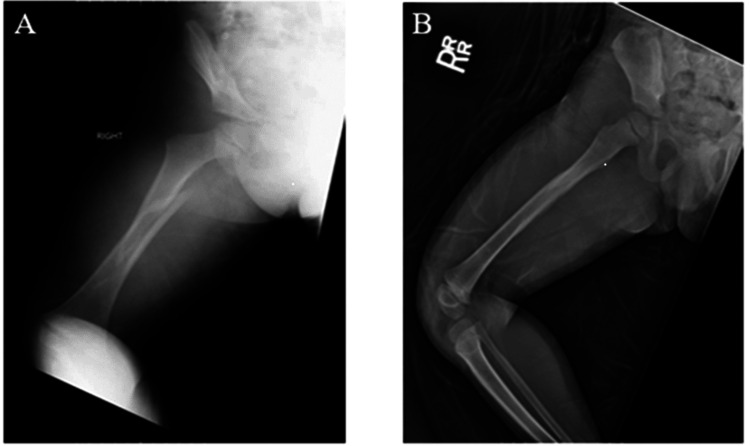
Initial imaging for case 4, including (A) AP and (B) lateral radiographs of the right lower extremity demonstrating a spiral fracture of the femoral shaft

Case 4 was a previously healthy and ambulatory three-year-old boy who was transferred from an outside ED following a fall from a high chair. He presented with the right lower extremity stabilized in a temporary posterior splint applied from the referring hospital. On examination, the patient's pain was stable, and he was neurovascularly intact in the right lower extremity. Radiographs demonstrated a displaced, right spiral midshaft femoral fracture with associated shortening (Figure 8A, 8B). The patient was taken to the operating room the following morning for the application of the DF2 brace under general anesthesia. Pre-manipulation radiographs demonstrated a 2 cm shortening with an associated mild varus deformity. Closed reduction was performed utilizing slight longitudinal traction combined with 30° of hip abduction and flexion. Before the final brace application, fluoroscopic imaging confirmed an improved reduction with 1 cm of residual shortening and mild valgus and recurvatum deformities. Protective stockinette was placed, followed by the secure fitting of the DF2 brace.

**Figure 9:**
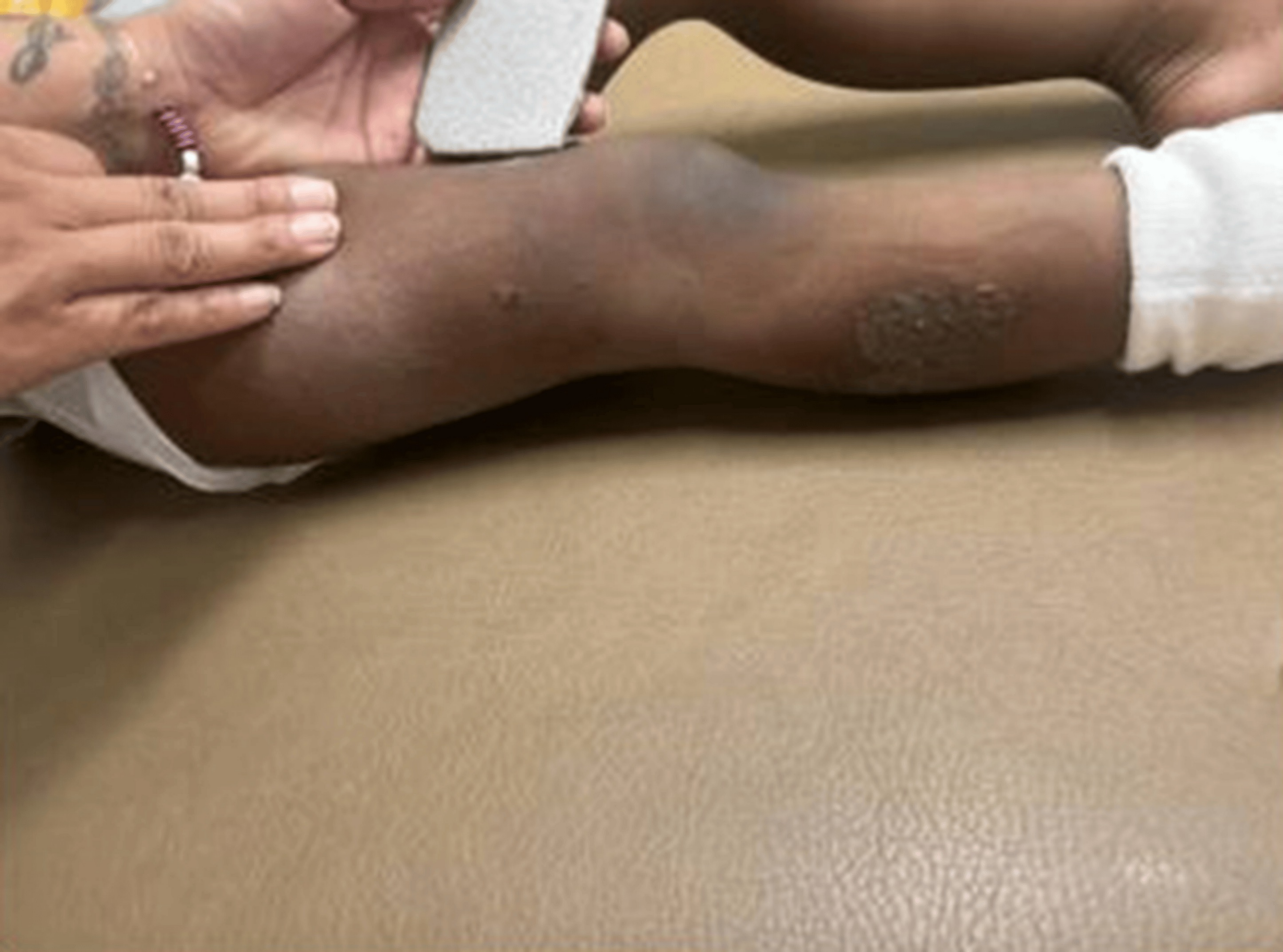
Image of case 4 at one-week follow-up that demonstrates a 2-3 cm superficial, open wound with associated tissue breakdown along the lateral right mid-calf

**Figure 10:**
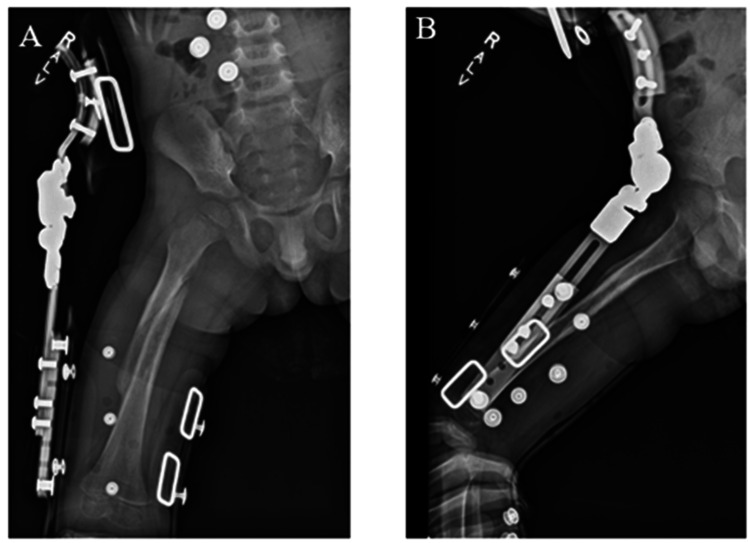
Final imaging for case 4, including (A) AP and (B) lateral radiographs of the right lower extremity demonstrating the healing of the spiral fracture of the femoral shaft

At the one-week outpatient follow-up, the patient's pain was successfully controlled using over-the-counter analgesics every six hours. The caregivers reported no skin concerns, and the overlying skin at the borders of the construct appeared intact. The soft tissue beneath the stockinette was not visualized during this appointment to avoid the premature manipulation of the brace early in the healing process. The patient returned for his scheduled evaluation at two weeks postoperatively. The caregivers noted that the patient had urinated into the brace and stockinette since his prior visit. Upon the removal of the construct, the physical examination revealed a 2-3 cm superficial, open wound with associated tissue breakdown along the lateral mid-calf (Figure 9). There was no associated purulent drainage or surrounding erythema present. Radiographs obtained at this visit not only demonstrated a slight increase in fracture shortening but also demonstrated early callus formation. The patient's family was provided with replacement stockinette and instructed to initiate a daily local wound care regimen with antibacterial soap, Betadine, and Mepilex silver dressing covered with Kerlix gauze. At the five-week postoperative follow-up, the calf wound healed appropriately, and radiographs confirmed stable fracture alignment (Figure 10A, 10B). The patient exhibited painless motion of the lower right extremity, and the DF2 brace was permanently discontinued.

Case 5

**Figure 11:**
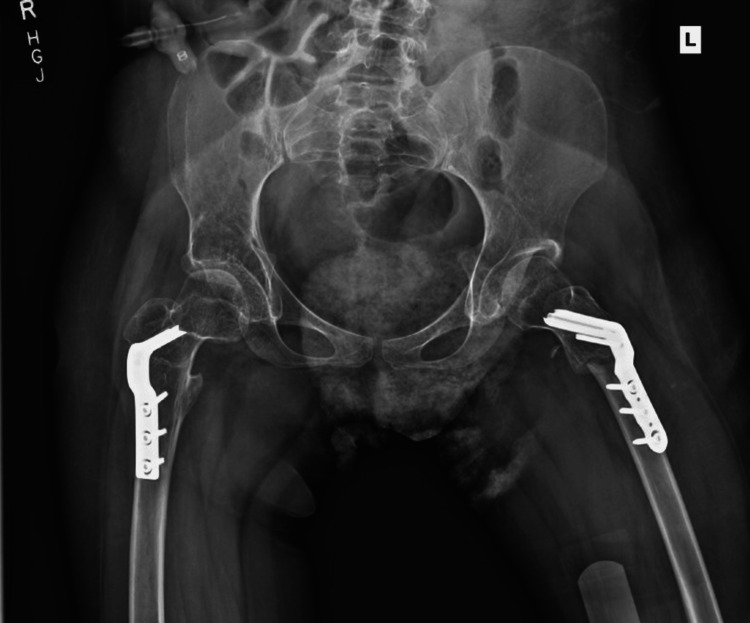
Postoperative radiographs of case 5, demonstrating the final alignment after bilateral hip adductor release and femoral varus derotation osteotomy

Case 5 was a 12-year-and-two-month-old girl with a medical history of developmental dysplasia of the hip (DDH) and Gross Motor Function Classification System (GMFCS) level V quadriplegic cerebral palsy who underwent an elective left hip adductor release and femoral varus derotation osteotomy (VDRO). To maintain postoperative immobilization and prevent hip flexion beyond 60°, a DF2 brace was applied in the operating room in conjunction with an abduction pillow (Figure 11).

On postoperative day 1, the inpatient orthotics team revealed that the proximal aspect of the brace was digging deeply into the soft tissues of the proximal thigh and groin. Additionally, the patient's caregivers expressed concerns regarding hygiene and skin care following voiding, noting that the patient's prior vesicostomy was already causing some issues. Because the brace could not be adequately modified in the inpatient setting, it was decided to discontinue the DF2 brace and to transition to a standard straight knee immobilizer in combination with an abduction pillow.

## Discussion

Traditional spica casting remains the standard treatment option for pediatric femoral fractures in patients under five years of age, but it carries well-documented limitations, including the need for general anesthesia, increased caregiver burden, hygiene difficulty, and the increased risk of skin complications such as pressure ulcers [4,7]. Prefabricated functional braces, such as the DF2, offer an alternative option for patients that limits anesthesia and provides greater independent mobility while maintaining equivalent fracture healing outcomes [10]. Overall, our study demonstrated that the DF2 brace achieved successful fracture healing with return to age-appropriate activity in all traumatic fracture cases while also identifying important considerations regarding skin surveillance, hygiene management, and patient selection.

An important observation in this series is that although functional bracing improved access for skin surveillance in our population, it did not eliminate the risk of pressure-related injuries. In our series, we observed open pressure injuries in two of our five patients, both located along the proximal lateral calf near the rigid distal border of the brace. This finding is consistent with the higher rate of superficial skin complications reported in the walking boot arm of a recent randomized controlled trial (RCT) comparing functional bracing with spica casting [4]. In our series, the mechanisms behind the skin ulcers were different between the two cases. In case 2, the injury appeared to be related to the direct mechanical pressure from the rigid brace border, whereas in case 4, the urine contamination of the stockinette likely created a moist environment that accelerated focal tissue breakdown. Notably, the authors of a randomized controlled trial (RCT) comparing removable boots to casting for toddlers' fractures of the tibia observed that early pressure sores in removable boots can be mitigated by routinely replacing a protective stockinette [11]. In case 1 of our series, proactive reinforcement with moleskin padding along the proximal thigh appeared to prevent skin complications, suggesting that targeted padding at high-risk contact points may serve as a protective factor. Importantly, the removable and adjustable nature of the DF2 brace allowed the skin complications in our study to be easily managed with local wound care in the outpatient clinical setting, avoiding a return to the operating room that would be required for spica cast changes.

Patient selection is another critical factor in the successful utilization of the DF2 brace. The current AAOS guidelines support prefabricated functional bracing for children with isolated diaphyseal femoral fractures [4]. Our case series demonstrated that for traumatic fracture cases, younger patients with acute diaphyseal or intertrochanteric fractures are able to achieve adequate bone healing and return to their previous level of activity. However, in case 5 of our series, we found a meaningful limitation of the current brace design. In the case of our 12-year-old with advanced cerebral palsy and an extensive surgical history in the pelvis and lower extremity, we found the proximal aspect of the brace impinging on the groin and a pre-existing vesicostomy site, which required a transition to a standard straight knee immobilizer and an abduction pillow. Similar experiences of postoperative immobilization after hip reconstruction in children with cerebral palsy have been highlighted in the literature, suggesting that atypical body habitus, spasticity, and pre-existing surgical sites in this population can limit downstream interventions [12]. Additionally, a recent RCT that compared spica casting with foam splinting after pediatric hip reconstruction found that foam splinting resulted in fewer complications and superior caregiver satisfaction, suggesting that alternative immobilization strategies may be more advantageous for complex reconstructive cases [13]. Overall, our study found that the DF2 brace is best applied to patients with acute diaphyseal femoral fractures, such as in young children, and that its application in patients with neuromuscular conditions or complex reconstructive hip surgery should be approached with caution.

Our study suggests that there are practical advantages to functional bracing for caregivers and patients. Spica casting has long been associated with significant caregiver burden, including difficulty with hygiene, transportation, and independent mobility factors. One observational study that compared family burden in children who received spica casting to flexible intramedullary nailing for acute femur fractures found significantly greater family impairments in the spica-casted group [3]. Furthermore, prior studies have demonstrated advantages of the DF2 brace with respect to caregiver burden and patient mobility. In the Andras et al. prospective RCT, patients in the bracing group had significantly less difficulty moving independently and were more than twice as likely to fit into their car seat when compared with their spica-casted counterparts [4]. These findings are clinically significant, as car seat incompatibility is a well-documented barrier to safe discharge [14]. Additionally, functional bracing has been shown to reduce hospital resource utilization. Stamatos et al. reported a mean reduction in length of stay (16.4 versus 26.2 hours, p=0.003) and total charges ($56,372 versus $78,892, p<0.001) for patients treated with prefabricated functional braces versus spica casting [10]. Finally, our series highlighted the importance of addressing language barriers during patient education. Our first case demonstrated that a non-English-speaking family required additional counseling regarding brace care and activity expectations, a challenge that has been recognized in the orthopedic literature as a likely contributor to poorer health outcomes [15,16].

An important finding in this series was the absence of a standardized protocol for postoperative brace management, which likely contributed to the variability of skin outcomes in our patients. Specifically, the frequency of stockinette changes and direct skin examinations differed among cases, with some patients having their entire brace and stockinette removed for skin checks while others did not have the brace removed to avoid disturbing the fracture reduction. For example, in case 4, this conservative approach may have allowed a moisture-related wound that was further contaminated with urine to progress into a complication that went undetected. Evidence-based pressure injury guidelines recommend that the skin beneath medical devices be kept clean, dry, and appropriately moisturized, with assessments performed at least twice daily when orthopedic devices such as casts are in use [3,4]. Establishing a uniform protocol regarding home adjustments, weight-bearing status, and the frequency of clinical soft-tissue checks is essential for optimizing favorable outcomes.

This study has several limitations. The retrospective nature and small sample size limit the generalizability of these findings and preclude meaningful statistics. Additionally, the follow-up period for most cases was relatively short, ranging from four weeks to three months, although this was sufficient to confirm bone healing and identify favorable outcomes and complications. The lack of a comparison group limits the ability to draw direct conclusions about the relative efficacy of DF2 versus spica casting. Future multicenter studies with larger cohorts and longer follow-up periods are required to establish evidence-based guidelines and formally compare the outcomes of functional bracing with traditional spica casting.

## Conclusions

This small case series suggests that the DF2 brace may be a feasible alternative to spica casting for select pediatric patients with acute femoral fractures, offering simplified hygiene access alongside reliable fracture healing in this case series. Based on this early experience, successful implementation appears to require careful patient selection, proactive skin protection, and a structured follow-up protocol to manage potential soft-tissue complications. Clinicians should counsel families on the need for stockinette management and the possibility of brace adjustments during the healing process. Given the retrospective design and limited sample size, these observations should be viewed as preliminary, and larger prospective studies are needed before broader conclusions can be drawn.
